# 707 Invasive Non-Candida Fungal Infections in Acute Burns: A 13-Year Review of a Single Institution

**DOI:** 10.1093/jbcr/irac012.261

**Published:** 2022-03-23

**Authors:** Arya A Akhavan, Feras Shamoun, Tomer Lagziel, Sohayla Rostami, Carrie A Cox, Carisa M Cooney, Charles S Hultman, Julie Caffrey

**Affiliations:** Johns Hopkins Hospital, North York, Ontario; Johns Hopkins University School of Medicine, 347 Morningside Ave, Ontario; Johns Hopkins University School of Medicine / Sackler School of Medicine, New-York Program, Tel-Aviv University, Rockville, Maryland; Johns Hopkins University, Baltimore, Maryland; Johns Hopkins Bayview Medical Center, Baltimore, Maryland; Johns Hopkins University School of Medicine, Baltimore, Maryland; Johns Hopkins University School of Medicine, Baltimore, Maryland; Johns Hopkins, Baltimore, Maryland

## Abstract

**Introduction:**

Burn patients have higher infection rates due to loss of the protective skin barrier. The past decade shows increased rates of burn infection with atypical invasive fungal organisms. After a recent trend of life-threatening atypical fungal burn infections at our hospital, we conducted this study to further characterize this.

**Methods:**

We identified patients admitted to our burn center from January 2008 to June 2021, who developed fungal non-*Candida* burn infections while admitted. We gathered demographic data, burn injury details, surgical treatment course, and fungal and bacterial infection data. Descriptive statistics were used to characterize the data and identify trends.

**Results:**

We identified 37 acute burn patients with atypical invasive fungal infections. Of these, 28 were infected with 1 species, and 9 were infected with multiple fungi.

Non-Candida fungi included *Aspergillus* (20), *Fusarium* (8), *Mucor* (6), and 11 other species. Three fungi were resistant to antifungals including amphotericin B. Other organisms included *Candida* (18), *Enterococcus* (13), *Pseudomonas* (9), and 19 other species. On average, patients were infected with 5 bacteria, had 13 antibacterial resistances, and required 6.5 antibacterials. There was one case of total-drug-resistant *Pseudomonas aeruginosa*. Every patient required Infectious Disease consultation. Eight patients became bacteremic and 1 became fungemic.

The average burn surface area was 35%. All patients required excisional treatment, with an average of 7 excisions, 7 coverage procedures, and 3.5 other procedures; 44% of patients required amputations for infection control. The most common complications were graft loss (39%), ventilator-associated pneumonia (28%), and death (28%). The median length of stay was 40 days (IQR = 89) for survivors and 28 days (IQR = 14) for nonsurvivors. All fatalities were from overwhelming polymicrobial infection. The average modified Baux score was 73 (± 28) for survivors and 102 (± 38) for nonsurvivors. All nonsurvivors had clean wounds without penetrating trauma.

**Conclusions:**

Burn patients with atypical invasive fungal infections have severe polymicrobial infections and extreme antibiotic resistance. Patients may require, or fail, treatment with last-line antibiotic therapy and amputation. Early Infectious Disease consultation and aggressive treatment is critical. Further research may elucidate risk factors and ideal treatment patterns.

