# Correction to: 707 Invasive Non-Candida Fungal Infections in Acute Burns: A 13-Year Review of a Single Institution

**DOI:** 10.1093/jbcr/irac042

**Published:** 2022-04-30

**Authors:** Arya A Akhavan, Feras Shamoun, Tomer Lagziel, Sohayla Rostami, Carrie A Cox, Carisa M Cooney, Charles S Hultman, Julie Caffrey

**Affiliations:** 1 Johns Hopkins University School of Medicine, Baltimore, Maryland; 2 Johns Hopkins University School of Medicine/ Sackler School of Medicine, New York Program, Tel-Aviv University, Rockville, Maryland; 3 Johns Hopkins Bayview Medical Center, Baltimore, Maryland

Arya A. Akhavan et al. *Journal of Burn Care & Research*, Volume 43, Supplement 1, April 2022, Pages S158–S159, https://doi.org/10.1093/jbcr/irac012.261

In the originally published version of this manuscript, there was a character error in the title that has been replaced with a colon, and author affiliation information was erroneously scrambled. These errors have been corrected.

